# Supplementary material for: Expert organisations with “challenging” and “complex” service users: Representation in English and Welsh autism charity reports and accounts
Source: PLoS One. 2025 Oct 24;20(10):e0335132. doi: 10.1371/journal.pone.0335132 (PMC12551846; doi:10.1371/journal.pone.0335132)
Supplement: S1 Table — (DOCX) [file pone.0335132.s001.docx]

**S1 Table: Findings related to actors other than Autistic people, the charity, and government.**

|  | **Actor** | **Theme** | **Sub-theme** | **National Autistic Society** | **Autism Initiatives Group** | **North East Autism Society** | **Autism Together** | **Ambitious about Autism** | **Prior’s Court Foundation** | **ESPA** | **Autism East Midlands** | **Autism Unlimited** | **Autism Anglia** | **Autism at Kingwood** | **Total** |
| --- | --- | --- | --- | --- | --- | --- | --- | --- | --- | --- | --- | --- | --- | --- | --- |
| Representation | Parents, carers & families | As the subject | As primary organisational stakeholders | x | x | x | x | x | x | x | x | x | x | x | **11** |
|  |  |  | Prioritised over service users | x | x |  | x | x | x |  |  |  | x |  | **6** |
|  |  |  | Speaking for service users | x | x | x |  | x | x | x | x |  |  | x | **8** |
|  | Donors | Enabler | Active and with agency | x | x | x | x | x | x |  | x | x |  | x | **9** |
|  |  |  | Enabling charities' work | x | x | x | x | x | x |  | x | x |  | x | **9** |
|  |  | As the subject | As organisational stakeholders | x | x | x | x | x | x | x | x | x | x | x | **11** |
|  | TARA  Readers | Enabler | Enabling charities' work | x |  |  |  | x |  |  | x | x |  |  | **4** |
|  | Trustees | As experts | Identified as professionals within photographs |  |  |  |  | x |  |  | x | x |  | x | **4** |
|  |  |  | As experts | x | x |  |  | x | x |  | x | x |  |  | **6** |
|  | Staff | As the subject | As heroes | x | x | x | x | x |  | x | x | x |  | x | **9** |
|  |  |  | As primary organisational stakeholders | x | x |  | x | x | x | x | x | x | x | x | **10** |
|  |  |  | As the focus of the work | x | x |  | x | x | x | x | x | x |  | x | **9** |
|  |  | As experts | As experts | x | x |  | x | x | x | x | x | x |  | x | **9** |
| Calls to action | Parents, carers & families | Engage with charity aims | Respond to policy | x |  |  |  |  |  |  |  |  |  |  | **1** |
|  |  |  | Work with/support the charity | x |  |  |  | x |  |  |  |  |  | x | **3** |
|  |  | Improve understanding | To become better trained or informed |  | x |  |  | x | x |  | x |  | x |  | **5** |
|  | Donors | Support the charity | Give funding or volunteer | x |  | x | x | x | x |  |  | x |  |  | **6** |
|  |  | Change practice | Become more inclusive | x | x |  |  | x |  |  |  |  |  |  | **3** |
|  | TARA  Readers | Support the charity | Give funding or volunteer |  |  |  |  | x |  |  |  |  |  |  | **1** |
|  |  |  | Engage with the charity | x |  |  |  | x |  |  |  | x |  |  | **3** |
|  | Trustees | Improve understanding | To become better trained or informed |  | x |  |  |  | x |  |  |  | x |  | **3** |
|  | Staff | Support the charity and personal development | To become better trained or informed |  | x |  | x | x | x | x | x |  | x | x | **8** |
|  |  |  | Ensure good performance |  |  |  | x | x | x |  |  |  |  |  | **3** |
|  |  |  | Take advantage of work benefits | x |  | x | x | x | x |  |  |  |  | x | **6** |
