# Supplementary material for: Expert organisations with “challenging” and “complex” service users: Representation in English and Welsh autism charity reports and accounts
Source: PLoS One. 2025 Oct 24;20(10):e0335132. doi: 10.1371/journal.pone.0335132 (PMC12551846; doi:10.1371/journal.pone.0335132)
Supplement: Attachment — Submitted filename: Response to Reviewers.docx [file pone.0335132.s003.docx]

| **Comment** | **Response** |
| --- | --- |
| Please ensure that your manuscript meets PLOS ONE's style requirements, including those for file naming. The PLOS ONE style templates can be found at https://journals.plos.org/plosone/s/file?id=wjVg/PLOSOne_formatting_sample_main_body.pdf and https://journals.plos.org/plosone/s/file?id=ba62/PLOSOne_formatting_sample_title_authors_affiliations.pdf | Style templates have been followed within this submission, including those for file naming. |
| Thank you for stating the following in the Competing Interests section:….Please confirm that this does not alter your adherence to all PLOS ONE policies on sharing data and materials, by including the following statement: "This does not alter our adherence to PLOS ONE policies on sharing data and materials.” (as detailed online in our guide for authors http://journals.plos.org/plosone/s/competing-interests). If there are restrictions on sharing of data and/or materials, please state these. Please note that we cannot proceed with consideration of your article until this information has been declared.  Please include your updated Competing Interests statement in your cover letter; we will change the online submission form on your behalf. | We can confirm that the statements in the Competing Interests section do not alter our adherence to PLOS One policies on sharing data and materials, and have added a statement to this effect. |
| Please amend your list of authors on the manuscript to ensure that each author is linked to an affiliation. Authors’ affiliations should reflect the institution where the work was done (if authors moved subsequently, you can also list the new affiliation stating “current affiliation:….” as necessary). | We have checked the affiliations. HA's affiliation is the University of Birmingham as stated, and we have listed their new affiliation with 'Current affiliation'. |
| Please include captions for your Supporting Information files at the end of your manuscript, and update any in-text citations to match accordingly. Please see our Supporting Information guidelines for more information: http://journals.plos.org/plosone/s/supporting-information. | We have updated our in-text citation for the supporting information S1 Table, and included a caption. |
| Please review your reference list to ensure that it is complete and correct. If you have cited papers that have been retracted, please include the rationale for doing so in the manuscript text, or remove these references and replace them with relevant current references. Any changes to the reference list should be mentioned in the rebuttal letter that accompanies your revised manuscript. If you need to cite a retracted article, indicate the article’s retracted status in the References list and also include a citation and full reference for the retraction notice. | We have reviewed our reference list. We have not cited any retracted articles. Most changes to the reference list are typographical; more substantial changes are the inclusion of eight additional references, as listed below: Abreu L. Autism: overview of policy and services. House of Commons Library Briefing Paper; 2024. Available from: https://commonslibrary.parliament.uk/research-briefings/cbp-7172/. Bottema-Beutel K, Kapp SK, Sasson N, Gernsbacher MA, Natri H, Botha M. Anti-ableism and scientific accuracy in autism research: a false dichotomy. Front Psychiatry. 2023;14: 1244451. CBM. What are models of disability 2025 [28th July 2025]. Available from: https://participation.cbm.org/why/disability-participation/models-of-disability. Czech H. Hans Asperger, National Socialism, and “race hygiene” in Nazi-era Vienna. Mol Autism. 2018;9(1): 29. Natri HM, Abubakare O, Asasumasu K, Basargekar A, Beaud F, Botha M, et al. Anti‐ableist language is fully compatible with high‐quality autism research: Response to Singer et al.(2023). Autism Res. 2023;16(4): 673-676. NICE: National Institute for Health and Care Excellence. Autism spectrum disorder in adults: diagnosis and management. 2021. Available from: https://www.nice.org.uk/guidance/cg142/resources/autism-spectrum-disorder-in-adults-diagnosis-and-management-pdf-35109567475909. O’Dell L, Bertilsdotter Rosqvist H, Ortega F, Brownlow C, Orsini M. Critical autism studies: exploring epistemic dialogues and intersections, challenging dominant understandings of autism. Disabil Soc. 2016;31(2): 166-179. Palermo MT. Preventing filicide in families with autistic children. Int J Offender Ther Comp Criminol. 2003;47(1): 47-57. |
| One thing missing was the year of the reports. The methods state that the reports were from the last five years, but no dates are provided in the text or the tables. | Table 2 includes the data of the Annual Reports in day/month/year format (column two). We have chosen not to repeat this information when we cite the reports, for brevity. |
| The authors use the acronym “TARA”, however, the meaning is not provided. | Thank you for this comment. On reflection, and paying attention to the accessibility of the language used in the document, we have chosen to write the name out in full, rather than using the 'TARA' acronym. |
| The interpretations of the visual analysis was less convincing since 6 of 11 reports had photos and in the majority of the photos it was not possible to identify who was represented within. | We feel that our interpretations of the photos considered is sufficiently robust, however we recognise that this less extensive than our interpretation of the text, precisely because of the unknown aspects that you refer to. We have added a reflection on this in our limitations section (p.31). |
| No, I viewed the link provided and it was not connected directly to the data (i.e., the TARA reports used for the analysis). I am uncertain how to access to the data on the website provided. | These reports are currently available on the register of charities published by the Charity Commission for England and Wales. Links from which the documents can be downloaded are given below. Please note that the Charity Commission regularly updates this information to publish the most recent five years' worth of reports; therefore the reports are not available in perpetuity and links may not be stable.  The National Autistic Society - https://register-of-charities.charitycommission.gov.uk/en/charity-search/-/charity-details/269425/accounts-and-annual-returns?_uk_gov_ccew_onereg_charitydetails_web_portlet_CharityDetailsPortlet_organisationNumber=269425 Autism Initiatives Group - https://register-of-charities.charitycommission.gov.uk/en/charity-search/-/charity-details/5090836/accounts-and-annual-returns?_uk_gov_ccew_onereg_charitydetails_web_portlet_CharityDetailsPortlet_organisationNumber=5090836 North East Autism Society - https://register-of-charities.charitycommission.gov.uk/en/charity-search/-/charity-details/1028260/accounts-and-annual-returns?_uk_gov_ccew_onereg_charitydetails_web_portlet_CharityDetailsPortlet_organisationNumber=1028260 Autism Together - https://register-of-charities.charitycommission.gov.uk/en/charity-search/-/charity-details/1007878/accounts-and-annual-returns?_uk_gov_ccew_onereg_charitydetails_web_portlet_CharityDetailsPortlet_organisationNumber=1007878 Ambitious About Autism - https://register-of-charities.charitycommission.gov.uk/en/charity-search/-/charity-details/3940203/accounts-and-annual-returns?_uk_gov_ccew_onereg_charitydetails_web_portlet_CharityDetailsPortlet_organisationNumber=3940203 Priors Court Foundation - https://register-of-charities.charitycommission.gov.uk/en/charity-search/-/charity-details/3950583/accounts-and-annual-returns?_uk_gov_ccew_onereg_charitydetails_web_portlet_CharityDetailsPortlet_organisationNumber=3950583 EASP - https://register-of-charities.charitycommission.gov.uk/en/charity-search/-/charity-details/1037868/accounts-and-annual-returns?_uk_gov_ccew_onereg_charitydetails_web_portlet_CharityDetailsPortlet_organisationNumber=1037868 Autism East Midlands - https://register-of-charities.charitycommission.gov.uk/en/charity-search/-/charity-details/517954/accounts-and-annual-returns?_uk_gov_ccew_onereg_charitydetails_web_portlet_CharityDetailsPortlet_organisationNumber=517954 Autism Unlimited Limited - https://register-of-charities.charitycommission.gov.uk/en/charity-search/-/charity-details/1000792/accounts-and-annual-returns?_uk_gov_ccew_onereg_charitydetails_web_portlet_CharityDetailsPortlet_organisationNumber=1000792 Autism Anglia - https://register-of-charities.charitycommission.gov.uk/en/charity-search/-/charity-details/3940357/accounts-and-annual-returns?_uk_gov_ccew_onereg_charitydetails_web_portlet_CharityDetailsPortlet_organisationNumber=3940357 Autism at Kingwood - https://register-of-charities.charitycommission.gov.uk/en/charity-search/-/charity-details/1041924/accounts-and-annual-returns?_uk_gov_ccew_onereg_charitydetails_web_portlet_CharityDetailsPortlet_organisationNumber=1041924 |
| Yes, however there are a few typos: Pg. 4 The last sentence has a direct quote – page number needed | Thank you for noticing these minor typographical errors. The manuscript has been proof read prior to this re-submission, including the missing page numbers. |
| Pg. 6 The last sentence ends with a comma and is incomplete. I also think that it is odd to state the findings this soon in the manuscript. | Thank you; we have revised this section to improve flow and have removed the summary of findings prior to the methods section. |
| Pg. 7 Part of the aim is to focus on the imagery, however, there was no background on how autism (or disability more broadly) imagery has been analyzed in the past. The background only focused on language/discourse. | Thank you for highlighting this; we have updated our literature review and background sections to demonstrate reference to both imagery and language. |
| Pg. 8 TARA is used but no definition is provided – it is in the abstract but never written out with (TARA) in the text. | Thank you for this comment. On reflection, and paying attention to the accessibility of the language used in the document, we have chosen to write the name out in full, rather than using the 'TARA' acronym. |
| Pg. 11 Typo – centering (not centring) | Again, thank you for reading with such a close eye and picking up on these typographical differences. In this instance, we have left as is - as centring is the British English spelling, and the rest of the paper is in British English. |
| The page numbering of the manuscript starts at page 1 again after the tables. | Thank you and apologies for all the page-numbering confusion. We have corrected the page numbering. |
| Pg. 8 – what is INGO? | Thank you; for ease we now refer to 'international development charities' |
| Pg. 9 (new page numbering) Second paragraph has a couple of direct quotes – page number needed. | Thank you; we have added page numbers for all direct quotes |
| Throughout the manuscript Disability is capitalized. Can the authors provide a note on why this is the case? Furthermore, Autism (not sure why?) and Autistic (which makes sense) are capitalized. Please justify the use of capitalizations. | Thank you for raising this important point. We have decided to uncapitalise ‘Disability’. To justify our continued capitalisation of Autism and Autistic, we have added a reference describing how this links to Autistic culture and identity: please see sentence included on page 3"In this paper, we capitalise Autism and Autistic as a marker of identity and culture [4]." |
| 1. In the introduction of the manuscript, it would be important for the authors to have a statement about how the differences of experiences by autistic people in communication, masking, sensory information processing, etc. vary across a spectrum. The heterogeneity of autism is an important aspect to articulate and will help situate the study. | Thank you for this point. We have briefly noted the history of dividing Autism, a singular diagnosis and the differences in lived experience among the Autistic community on page 4. |
| 2. I appreciate the clear articulation of the medical, charity, social and psychosocial model of disability. It was unclear if the authors were taking a social or psychosocial model approach in their analysis. There is alignment with findings to the medical model but not the other models – not even the charity model. How does this research add to our understanding of these models of disability? | We have added to both the Discussion and Conclusion to demonstrate that our findings fit within both the medical and charity models of disability and to address both this and a further comment below. Please see the additional paragraphs on pages 33-4: "Our findings therefore demonstrate that the visual and text-based discourse constructed by these charities is situated within the medical and charity models of disability – both of which individualise disability, and frame disabled people as ‘victims’, lacking in agency. As Shakespeare [11] demonstrated, responsibility for the ‘problem’ of Autism is placed on Autistic people. In contrast, the charity is portrayed as active and expert, and as “knowing what is good for” [55] Autistic people. The social model of disability, based on a rights-based approach that seek to address societal barriers to the emancipation of disabled people, is not reflected in this discourse.  Further, in this paper we demonstrate the value of applying a lens to UK charities which has hitherto largely focused on the field of international development charities. We find similar themes here that have been more widely demonstrated within the international development discourse, but have not previously been robustly evidenced within the discourse of English and Welsh charities working with minoritised or marginalised groups - including Autistic people - in countries such as the UK. Our findings also show how the discourse constructed by the Autism charities studied here reflects the “non-disabled saviour” trope that has been found to be common in popular culture [56], but has again not thus far been studied extensively within other discourses. As such, we believe this paper contributes to a deeper understanding of the construction and operationalisation of the medical and charity models of disability in contemporaneous English and Welsh charity discourse." |
| 3. Relatedly, although the author’s cite Yergeau’s book, I am surprised that the study is not framed using Critical Autism Studies approach, which aligns with much of the analysis: challenges medicalization and deficit model, autistics as agents and producers, examines how institutions like the charities under investigation shape the understanding and lives of autistic people, reflexive approach, etc. | Thank you for this suggestion. We do work within a critical paradigm aligned to the social model of disability, and agree that it would be sensible to reference Critical Autism Studies as part of our approach. We have included this reference at three points within the revised manuscript: within the aim on page 9; within our conceptual and theoretical approach on page 11; and when describing our analytic process on page 13. |
| 4. Pg. 5 – Can the authors elaborate on what is meant by “altruistic’ filicide”? | We have added the definition on page 6: "(altruistic filicide refers to the idea of “‘murder committed out of love’”, which in cases involving disabled people can “dangerously overlap the idea of mercy killing”(p.47). [23]" |
| 5. Pg. 6 – in the brief review of literature, the authors may also want to add that charity discourses reflect the priorities of the charities and the work that they do, what they fund, the programs they create/sponsor, etc. thus the discourses reflect the actions they are taking. | Thank you for this important reflection; we have added a comment on page 7. |
| 6. Pg. 6. The manuscripts states that there is an “impact and influence of Autism charities in the UK”. Given this statement, the manuscript should provide some information about the influence/impact of autism charities in the UK. What percentage of funding (compared to direct billing by NHS and other state/government programs) is from charities? Do charities cover care only (as opposed to research?)? Some contexts here is needed to show how these charities really matter when it comes to financial investments (and the discourses they carry about autistic people). | Thank you; we have sought to demonstrate the impact and influence of Autism charities on page 6, and have added further information to this section. |
| 7. Although the photos were unequally represented in the reports and most did not identify who was in the photo (autistic, care giver, etc.), I am curious if age and gender were also coded, especially given the focus on childhood autism and over representation in boys. In the US, race and ethnicity are also underrepresented in the media/culture/discourses. This is one area that I found missing even in the discursive analysis. | Thank you for this comment. Given the lack of information about who is represented in the photographs, coding - for gender, age, and race - would have involved an element of 'guesswork' and therefore was not included. |
| 8. Pg. 1 (new page numbering) – in the analytic findings, the authors mention “non-human actors” – I am curious what is meant by this phrase since all the actors listed are human entities (even charities). Machines, technologies, diagnostic tools, policies, etc. would be non-human, but this is not what was analyzed. One way to consider this is that the reports themselves serve as a non-human actor given the discursive work that they do beyond the human production of the reports? I guess the use of non-human in this sentences seems out of place. | Thank you for this comment. For clarity, we have removed this phrase. |
| 9. Pg. 8 – Discussion – this is related to the previous comment about the UK context – the authors state “Government is primarily portrayed as a barrier to the effective……..” My question as a reader – why is this important in the context of the UK, which has National Health Care? Knowing more about the types of national service programs dedicated to autism through NHS or other governmental programs would be helpful. Furthermore, when stating “known deficiencies in public services when used by Autistic people” – are the authors referring to services outside of the charities and if so, can they provide an example? | Thank you for this point; unfortunately the UK NHS does not comprehensively provide such services. As described earlier in the paper, the UK has a mixed economy of health in which servies are provided by both charities and the state. Services - including health and social care - are therefore provided both directly through state bodies, through state funding of charities, and through charities raising funding to provide independent services. In this section, we are referring to government being seen as a barrier for the effective implementation of services by charities. We have made this clearer in the text. Furthermore, we add additional information on p.31 regarding these known deficiencies in public services |
| 10. Pg. 9 – Discussion - I appreciate the critique of PBS, However, there are cases where PBS can be helpful for autistic people, especially if they are at risk of injuring themselves. This is extreme, of course, but must also be acknowledged. The authors critique to lack of heterogeneous representation of autism in the TARA and should equally be reflexive of the heterogeneity of different approaches that autistic people have benefited from to have greater voice and representation. There is a fine line that the authors should acknowledge. | Thank you for this point. Whilst you correctly point out that PBS and ABA approaches *can* be used to prevent self injury or other significant areas of danger (eg: knowing how to respond to a police officer as a Black teenage boy, when you might otherwise be shot), this is not the primary use of these therapies, many of which require Autistic children and adults to behave in neurotypical ways including saying exact phrases, giving eye contact etc with comfort items withheld until the behaviour has been performed to the clinicians satisfaction, when there is no risk of injury or harm from performing these tasks in an Autistic way. As such, we have chosen not to acknowledge the rarer appropriate use of these approaches (to prevent harm). |
| 11. Pg. 10 – Discussion – I would have liked to learn more about the systemic barriers that Autistic people face? How does this analysis support these barriers? | As above, we have added some more information about these systemic barriers on page 31, and refer again to this in the conclusion. |
| 12. As stated before, these findings are not that surprising. To help add to the significance of these findings, can the authors say something about how autism is different than other disabilities as it relates to the findings? Or is this just another example of how differences are viewed as disabling in medical models of disability? In other words, how might the analysis of autism bring new insight into the pathologization of disability? Charity model of disability? Critical Autism Studies? | Thank you for this comment, which has enabled us to reflect on our contribution to the literature. While we agree that our findings may be unsurprising to some readers, others may be surprisesd by the findings. We therefore feel that this paper adds value to the debate, by providing the first (we believe) robust evidence analysing the discourse of English and Welsh Autism charities. As above, this is reflected in our additional paragraphs included on pages 33-4. |
| 13. Limitations – one question that came up is the target audience of these reports? Who is the target audience and how might this shape the discourse? Are they meant for broad general audience? Government funders? Autistic people and their families? Clinical professionals? | Thank you; we have reflected on this and added additional text in our limitations section. |
